# A Genuine Hermaphrodite

**Published:** 1868-12

**Authors:** Henry N. Avery

**Affiliations:** Poughkeepsie, N. Y.


### A Genuine Hermaphrodite.

BY HENRY N. AVERY, M. D., POUGHKEEPSIE, N. Y.

The following is such a wonderful case, and being as near a hermaphrodite as anything can be, notwithstanding the evidence that nothing of the kind can exist, I report it for the novelty of the case, rather than the operation:

August 6th, 1868, Christie Ann C., called upon me for advice, giving the following answers to my questions. After stating that she was a native of Nova Scotia, and had just arrived in this city to see a sister living here, and seek surgical aid in the States; unmarried; twenty-four years of age; five feet ten inches high; enjoying comparatively good health; occupied during the past two years in teaching school, and that she had a *growth* upon her privates.

From observations, I discovered that she possessed a deep, coarse voice, a masculine frame and face; in fact, resembling an ordinary coarse woman.

After a careful examination, to my surprise I found the following to exist: the mammæ were undeveloped; the clitoris, resembling a penis in flaccid state, was two inches long, and half an inch in diameter, with well developed gland and foreskin. No orifice was discovered. A vagina two inches deep, well formed, existed, but a close examination per rectum and bladder could not discover any trace of a uterus, the meatus urinarius and vestibule were perfect; the right labium majorum was quite natural and of usual size; the labia minora were traceable, but in the folds of the left labium there appeared a large pendant tumor, resembling the left *testicle* of a man, with a well developed scrotum of usual size, of some four inches in length, resembling in every respect the scrotum. Tracing what appeared to be the cord up, I found it made its exit from the external abdominal ring, and having every indication of a spermatic cord; the epididymis appeared to be natural; in fact, everything resembled a *testicle.*

She stated that she felt some sexual desire, and that every morning for the past six years, she had vomited, on rising from bed, a small quantity of blood. To my question as to how long the tumor had existed, she stated that she had noticed nothing until she was ten years of age.

Her object in coming to me was, she said, to see if I would remove the tumor, as it annoyed her. The physician at home, the only one she had ever shown it to, stated that he could do nothing for her.

Being placed in somewhat of an embarrassing position, in discovering so much more than I expected to find, I resolved to call a consultation, to see if my diagnosis of a *testicle* would be confirmed Accordingly Drs. J. S. P. Lord, E. H. Parker, and my brother, Dr. E. W. Avery, all of this city, were called in, when they all agreed that it resembled in every respect a *testicle*, but the case being so extraordinary that they could not form any diagnosis, but advised an operation.

With the assistance of Dr. Lord and Dr. E. W. Avery, I proceeded to perform the operation, by removing the tumor by the usual process of castration, by making an incision of some five inches in length, so as to expose the cord, which was found with three arteries that were ligated, and several smaller ones, a large nerve, vein, etc.; severing the cord, the retraction was the same that might be expected in performing the operation upon a man; the tumor was then dissected out, the wound partially closed, and the patient placed in bed.

After removal, the tumor was examined by Dr. Lord, Dr. E. W. Avery and myself, with a microscope magnifying 350 times, when cellular structure and convoluted tubes were visible, with rudimentary spermatozoa; in fact, it was declared a *testicle.*

Mounted specimens of the tubes for the microscope have been prepared, and photographs of the woman will be preserved.

This being the only case, I believe, on record, where a *testicle* has been discovered in a *woman*, it will naturally interest many. The *fact* can now be settled, that such a thing as a hermaphrodite has existed.—*Medical and Surgical Reporter.*

